# Optical Nanoantennas for Photovoltaic Applications

**DOI:** 10.3390/nano11020422

**Published:** 2021-02-07

**Authors:** Francisco Duarte, João Paulo N. Torres, António Baptista, Ricardo A. Marques Lameirinhas

**Affiliations:** 1 Department of Electrical and Computer Engineering, Instituto Superior Técnico, 1049-001 Lisbon, Portugal; joaoptorres@hotmail.com (F.D.); joaotorres@tecnico.ulisboa.pt (J.P.N.T.); baptista@tecnico.ulisboa.pt (A.B.); 2 Instituto de Telecomunicações, 1049-001 Lisbon, Portugal; 3 Academia Militar, Av. Conde Castro Guimarães, 2720-113 Amadora, Portugal; 4 Centro de Investigação, Desenvolvimento e Inovação da Academia Militar, Av. Conde Castro Guimarães, 2720-113 Amadora, Portugal

**Keywords:** nanoantennas, optics, optoelectronic devices, photovoltaic technology, rectennas

## Abstract

In the last decade, the development and progress of nanotechnology has enabled a better understanding of the light–matter interaction at the nanoscale. Its unique capability to fabricate new structures at atomic scale has already produced novel materials and devices with great potential applications in a wide range of fields. In this context, nanotechnology allows the development of models, such as nanometric optical antennas, with dimensions smaller than the wavelength of the incident electromagnetic wave. In this article, the behavior of optical aperture nanoantennas, a metal sheet with apertures of dimensions smaller than the wavelength, combined with photovoltaic solar panels is studied. This technique emerged as a potential renewable energy solution, by increasing the efficiency of solar cells, while reducing their manufacturing and electricity production costs. The objective of this article is to perform a performance analysis, using COMSOL Multiphysics software, with different materials and designs of nanoantennas and choosing the most suitable one for use on a solar photovoltaic panel.

## 1. Introduction

In the last decade, the advances in the nanoscale dimension enabled the development of new devices, such as nanoantennas or optical antennas, due to the emergence of a new branch of science known as nanooptics, which studies the transmission and reception of optical signals at the nanoscale. These devices have been the object of intense research and development activity, with the goal to reach the captivating possibility of confining the electromagnetic radiation in spatial dimensions smaller than the wavelength of light.

The transmission through a metal plane with subwavelength-sized holes can be drastically increased if a periodic arrangement of holes is used. This phenomenon is widely known as Extraordinary Optical Transmission [1]. The usage of nanoantenas with apertures smaller than the light wavelength can locally enhance light–matter interaction. Thus, nanoantennas are devices that have the ability to manipulate and control optical radiation at subwavelength scales.

Nanoantennas are a nanoscale version of radio-frequency (RF) or microwave antennas. However, throughout this article it will be proven that in the process of sizing the nanoantennas, it will not be enough to reduce the size of the RF antennas to the optical domain, mainly because of the unique material properties of metals that influence the behavior of antennas at the nanoscale: the existence at the interface between metals and dielectrics of surface plasmon-polariton electromagnetic waves, which gives rise to resonant effects not available at RF [1,2].

The use of optical antennas for solar energy harvesting has received significant interest as they represent a viable alternative to the traditional energy harvesting technologies. Economical large-scale fabrication of nanoantenna devices would support applications such as building integrated photovoltaics and supplementing the power grid [3].

## 2. Rectenna System for Solar Energy Harvesting

The nanoantenna itself does not convert the collected AC current into DC current, and so it needs to be complemented with a rectifying element. The whole structure is commonly referred to as a rectenna [4].

A rectenna is a circuit containing an optical antenna, filter circuits, and a rectifying diode or bridge rectifier for the conversion of electromagnetic energy propagating through space (solar energy) into DC electric power (through the photovoltaic effect).

**Figure 1:**
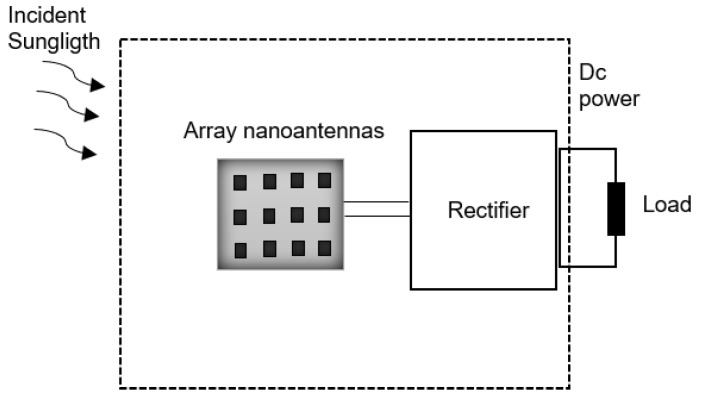
Representation of the nano-rectenna system (adapted from the work in [5]).

A schematic representation of a nano-rectenna system is depicted in Figure 1.

First, electromagnetic radiation is collected by the nanoantenna device. However, the output obtained from a single nanoantenna element is not enough to drive the rectifier and to provide DC power to an external load. The efficiency of a single optical antenna is generally low and its functionality is limited. Therefore, nanoantennas are arranged into arrays to increase their signal. The total field captured by the array is the addition of the fields captured by each nanoantenna [6].

The AC current generated in the nanoantenna arrays is collected and rectified into DC current by the rectifier system. This system has different rectifiers whose outputs can be DC coupled together, allowing arrays of nanoantennas to be networked to further increase output power [3].

As optical radiation requires high-speed rectification, high frequency metal–insulator–metal (MIM) diodes—also known as tunneling diodes—are commonly used for this purpose.

According to Moddel and Grover, the MIM diodes must have three key characteristics in order to have an efficient rectifier system [6]: high responsivity, that is, a measure of the rectified DC voltage or current as a function of the input radiant power; low resistance, in order to have a good impedance matching between the antenna and the diode; and asymmetry in the I–V curve, so the diode must have asymmetric characteristics for the rectenna be operated without applying an external DC bias.

Examples of material combinations used for diode rectifiers include Ni/NiO/Ni, Nb/Nb2O5/Pt, Nb/TiO2/Pt, Cu/TiO2/Pt, Nb/MgO/Pt, and Nb/Al2O3/Nb [6].

## 3. Experimentally Studied Nanoantenna Materials and Designs

A large variety of nanoantenna geometries has been researched for multiple potential applications. Currently, nanoantennas structures are mainly made of plasmonic materials, i.e., specially designed metal (usually gold or silver) nanoparticles with unique optical properties. Plasmonic materials exhibit strong light absorption in the visible region of the spectrum [4].

**Figure 2:**
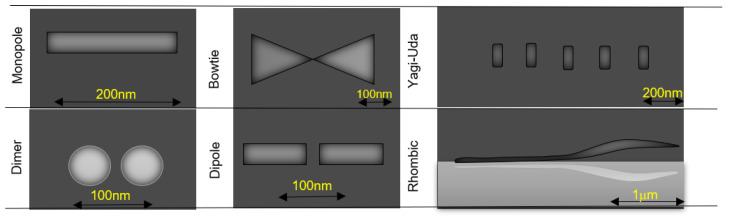
Main types of plasmonic nanoantennas (adapted from the work in [7]).

The advances in the manufacturing techniques allowed the construction of different formats of nanoantennas. The main types of plasmonic nanoantennas that have been proposed and investigated experimentally are represented in Figure 2.

### 3.1. Plasmonic Monopole Nanoantenna

The most basic type of plasmonic nanoantenna, a monopole, is a single metallic nanoparticle that can enhance the electromagnetic field strength in its surrounding area upon excitation of plasmon resonances. Monopole nanoantennas have advantages over other geometries, because they are easier to engineer and are well isolated from interference due to the ground plane. Their characteristics are dependent on the shape, size, material, and dielectric environment of the nanoparticle [7].

Another great utility of the monopole optical antenna is when it is integrated in a Near-field Scanning Optical Microscopy (NSOM), to be used as a near-field probe for measurements [8]. An effective nanoantenna can be used in spectroscopy: it needs to interact strongly with incident electromagnetic radiation in order to measure its intensity.

### 3.2. Plasmonic Dipole Nanoantenna

Dipole configurations are widely used in radio frequency and microwave ranges. Therefore, it is not a surprise that analogs of such antennas also appeared in the optical range. This type of optical antenna is widely used in near-field optical probes, just like the monopole. The dipole optical antenna is constituted either by dimers or two monopoles separated by a small space (gap). Usually, there is a high field confinement in the gap between the two metallic nanoparticles [7].

The design of plasmonic nanoantennas may rely on the same principles used in RF antennas. For example, the length of the dipole RF antenna is approximately half the wavelength of the incident radio waves, whereas the length of the dipole plasmonic nanoantenna is smaller than the wavelength, λ, of incident light in free space [9].

### 3.3. Plasmonic Bowtie Nanoantenna

Another typical structure is the bowtie nanoantenna, consisting of two triangular shape nanoparticles aligned along their axes and forming the feed gap with their tips. These optical antennas are a variant of the dipole nanoantennas. Such geometry ensures a wider bandwidth together with large field localizations in the feed gap compared to the straight dipole.

The bowtie topology is considered to be one of the most efficient nanoantenna geometries for solar energy harvesting. According to Sen Yan [5], in their study it is shown that the bowtie topology can increase the total radiation efficiency and rectenna efficiency compared to the straight dipole by a considerable 10%.

### 3.4. Plasmonic Yagi-Uda Nanoantenna

RF Yagi–Uda type antennas are usually used to receive TV signals from remote stations, due to their high directivity. Their plasmonic counterparts consist of a reflector and one or several directors.

Yagi–Uda optical antennas can be useful in many applications: in wireless communications, in the fields of biology and medicine, in nanophotonic circuits, in quantum information technology, in data storage (as an optical chip), in photodetectors, and in photovoltaic (PV) systems.

### 3.5. Plasmonic Spiral-Square Nanoantenna

**Figure 3:**
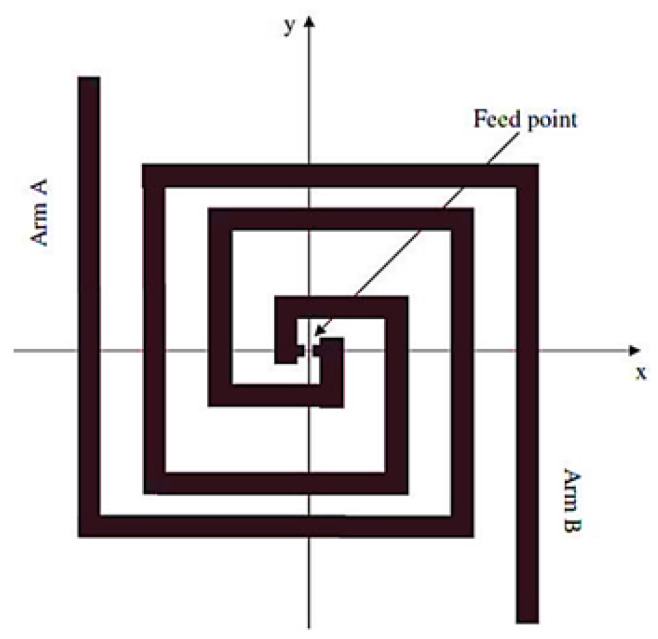
Geometry of a square-spiral nanoantenna (sourced from the work in [10]).

This design of nanoantenna allows the electromagnetic radiation to be harvested in one specific point in its structure—the gap (feed point) between two metallic arms, as presented on Figure 3. Thus, this topology has a wider angle of incidence exposure in comparison to other formats, which makes it an ideal geometry for solar energy harvesting.

They also demonstrate a high directivity that can be further improved by increasing the number of arms.

### 3.6. Dielectric Nanoantennas

A new research direction of optical antennas has recently been suggested with the introduction of dielectric nanoantennas. Optical antennas constructed with dielectric materials have several advantages over their metallic counterparts due to unique features not found in plasmonic nanoantennas [4].

Dielectric nanoantennas are fabricated from optically transparent materials that have low dissipative losses at optical frequencies. Unlike gold or silver, dielectric nanoantennas are usually made from silicon nanoparticles which are widely used in nanoelectronics to fabricate transistors and diodes. Furthermore, silicon has a high permittivity and exhibits very strong electric and magnetic resonances at the nanoscale, and thus improves radiation efficiency and antenna directivity, expanding the range of applications for nanoantenna structures [4,11].

**Figure 4:**
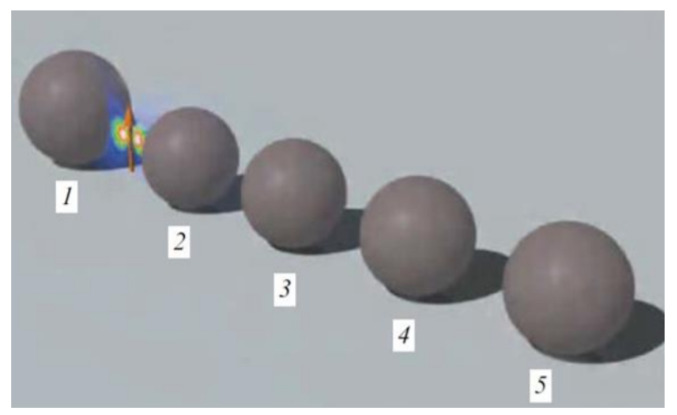
3D view of an all-dielectric optical Yagi–Uda nanoantenna, consisting of the reflector 1 of the radius Rr = 75 nm, and smaller director 2–5 of the radii Rd = 70 nm (adapted from the work in [7]).

The authors of [11] used silicon nanoparticles to demonstrate the performance of all-dielectric nanoantennas. They have analyzed an all-dielectric analog of the plasmonic Yagi–Uda nanoantenna consisting of an array of nanoelements: four directors and one reflector particle made of silicon. In this type of structure, the optimal performance is obtained when the director nanoparticles sustain a magnetic resonance and the reflector nanoparticle sustains an electric resonance [4]. schematic representation of this antenna is shown in Figure 4.

The dipole source is placed equally from the reflector and the first director surfaces at the distance D. The separation between surfaces of the neighboring directors is also equal to D.

The operational regime of a dielectric Yagi–Uda nanoantenna strongly depends on the distance between its elements. According to Krasnok [11], in their study it was verified that the radiation efficiency of the dielectric Yagi–Uda nanoantenna slowly decreased with decreasing distance between its elements, while the radiation efficiency of a plasmonic antenna of similar design and dimensions was greatly affected by the decrease in distance between particles. This is due to increased metal losses caused by proximity of adjacent metallic nanoparticles.

However, for larger separation distances, D, the radiation efficiencies of both types of nanoantennas were very identical. Although dissipation losses of silicon are much smaller than those of silver, the dielectric particle absorbs the EM energy by the whole spherical volume, while absorption only occurs at the surface of metallic particles. As a result, there is no substantial difference in the performance of these two types of nanoantennas for relatively large distances between its elements.

To sum up, based on the results of this study, a conclusion could be made that all-dielectric nanoantennas demonstrate major advantages over their metallic counterparts: much lower Joule losses and strong optically induced magnetization [11].

### 3.7. Aperture Nanoantennas

There is another type of optical antenna that is interesting for the topic of this article: aperture optical antennas. Light passing in a small aperture is the subject of intense scientific interest since the very first introduction of the concept of diffraction by Grimaldi in 1665 [12].

**Figure 5:**
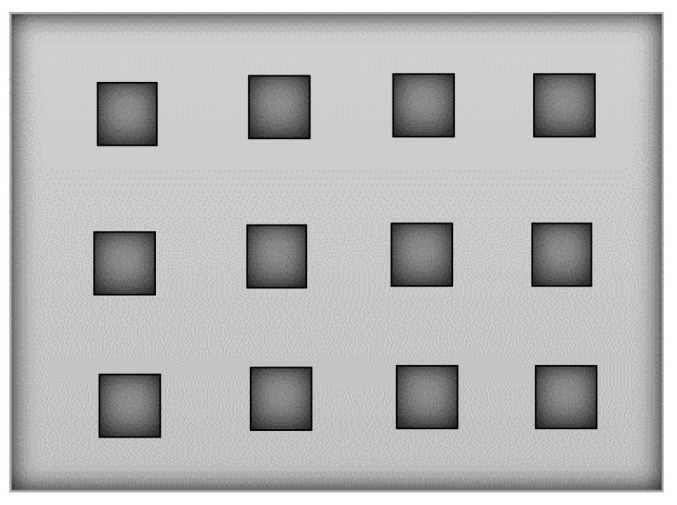
Schematic view of 200 nm diameter aperture arrays with 1 um period (adapted from the work in [12]).

The first theory of diffraction due to a slit, that is much less than the light wavelength, in a thin metal layer was developed by Bethe. This theory predicted that the power transmitted by the slit would decrease as the slit diameter decreased relative to the wavelength of the EM radiation. This theory proved to be incorrect when Ebbesen, in 1998, observed the extraordinary optical transmission phenomenon (EOT) [1]. The EOT is an optical phenomenon, in which a structure containing subwavelength apertures transmits more light than might naively be expected. Ebbesen et al. observed that when focusing a light beam in a thick metallic film where there was a subwavelength hole array, a large increase of incident electromagnetic wave transmission occurs, i.e., a periodic array of subwavelength holes, as presented in Figure 5, transmits more light than a large macroscopic hole with the same area as the sum of all the small holes [1,2,13,14,15,16,17,18,19].

This discovery would be fundamental, as it not only allowed great technological developments during the last decade, but also allowed a better understanding of the diffraction by small slits in relation to the light wavelength [20,21].

According to Wenger, there are three main types of aperture antennas [12]: single subwavelength aperture, single aperture surrounded by shallow surface corrugations, and subwavelength aperture arrays.

## 4. Surface Plasmon Resonance

As referred in the introduction, incident light on the optical antenna causes the excitation of free electrons in metallic particles. More precisely, EM waves induce time-varying electric fields in the nanoantenna that apply a force on the gas of electrons inside the device, causing them to move back and forth at the same frequency range as the incoming light. This phenomenon is known as surface plasmon. At specific optical frequencies the nanoantenna resonates at the same frequency as the incoming light which enables the absorption of the incoming radiation [4,15,16,17,18,19].

It should be taken into account that, at optical frequencies, metals do not act as perfect conductors: their conductivity changes dramatically, and so they are unable to respond to the time-varying electric field immediately. The wave propagation within the material is affected, which means that the penetration of EM radiation into metals can no longer be neglected.

Thus, at optical frequencies an antenna no longer responds to external wavelength but to a shorter effective wavelength that depends on the material properties [20].

EM radiation penetrates the metal of the nanoantenna and gives rise to oscillations of the free-electron gas. These electron oscillations can give rise to plasmon resonances, depending on the size, shape, and index of refraction of the particle as well as the optical constants of its surrounding [21].

When these oscillations are optimized, i.e., when the metal structure is sized to achieve the resonance condition, it is called Surface Plasmon Resonance (SPR). It is also important to mention that there are two types of surface plasmons [15,16,17,18,19,22]: Surface Plasmon Polariton (SPP), when the EM waves strike a metallic film and are confined to the surface of this film, and Localized Surface Plasmon (LSP), when the coupling is made with a metal nanoparticle with a diameter much smaller than the incident wavelength.

Surface plasmons are highly confined energy fields made by the oscillation of electrons on the surface of nanoantennas. When a metallic nanoparticle is illuminated by light, surface plasmons will be coupled with the photons of incident light in the form of a propagating surface wave [23].

SPPs are infrared or visible frequency EM waves trapped at or guided along metal–dielectric interfaces [24]. This coupling of plasmons—either SPPs or LSPs—and photons results in charge oscillation in the visible and infrared regimes depending on the metal used. SPPs are shorter in wavelength than the incident light (photons). Therefore, SPPs provide a significant reduction in effective wavelength and have tighter spatial confinement and higher local field intensity [24].

Recent development of nanofabrication techniques enabled construction of a variety of metal structures at the subwavelength scale and opened the research area called plasmonics, a subfield of nanophotonics studying the manipulation of light coupled to electrons at the nanoscale.

The properties of optical antennas are still under the intensive study and so research efforts to relate plasmonics with subwavelength optical antenna are in a developing stage [23].

## 5. Efficiency

The radiation efficiency of nanoantennas is a key parameter for solar energy harvesting. It is the first factor in the total efficiency product by which nanoantennas can convert incident light to useful energy. This efficiency depends directly on the type of metal used as conductor and the dimensions of the nanoantenna [6].

The main advantage of this type of technology in comparison to the conventional solar photovoltaic cells is its far greater efficiency by which the transformation of electromagnetic energy into DC electric power is performed. Typical efficiencies for traditional silicon cells are in the order of 20%, whereas nanoantennas go from a stunning 70% for silver nano-dipoles [25] to a more realistic 50% for aluminum dipoles [26]. Most solar radiation is in the visible and infrared (IR) wavelength region, and so nanoantennas need to be designed for this part of the spectrum, with the aim of being an alternative to conventional solar photovoltaic cells.

The total efficiency of a rectenna consists of two parts: (1) the efficiency by which the light is captured by the nanoantenna and brought to its terminals, also known as radiation efficiency, $\eta_{total}^{rad}$, and (2) the efficiency by which the captured light is transformed into low frequency electrical power by the rectifier, $\eta_{total}^{mat}$.

According to Kotter, the total radiation efficiency could be given by expression 1 [25], where λ is the wavelength of the incident light and the upper and lower integration limits $\lambda_{start}$ and $\lambda_{stop}$ should cover the optical bandwidth for the solar energy harvesting.

(1) $$\eta_{total}^{rad}=\frac{\int_{\lambda_{start}}^{\lambda_{stop}}P_{inc}\left(\lambda \right)\eta^{rad}\left(\lambda \right)d\lambda }{\int_{\lambda_{start}}^{\lambda_{stop}}P_{inc}\left(\lambda \right)d\lambda }$$

Furthermore, $P_{inc}\left(\lambda \right)$ is a function of the wavelength that follows Planck’s law for black body radiation according to expression 2, with T being the absolute temperature of the black body that in this case is the temperature of the surface of the sun, h the Planck’s constant, c the speed of light in vacuum, and k the Boltzmann constant.

(2) $$P_{inc}\left(\lambda \right)=\frac{2\pi hc^{2}}{\lambda^{5}}\frac{1}{e^{\frac{hc}{\lambda kT}}-1}$$

$\eta^{rad}$ is the radiation efficiency of the antenna as a function of the wavelength that is given by expression 3, where $P^{rad}$, $P^{inj}$, and $P^{loss}$ are the radiated power, the power injected at the terminals, and the power dissipated in the metal of the nanoantenna, respectively.

(3) $$\eta^{rad}=\frac{P^{rad}}{P^{inj}}=\frac{P^{rad}}{P^{rad}+P^{loss}}$$

In order to generate DC power in the load, a rectifier is connected to the input port to rectify the current flowing in the antenna’s structure that oscillates around hundreds of THz. Like the total radiation efficiency, it is also possible to define the total matching efficiency as described on expression 4, where $\eta^{mat}$ is the matching efficiency of the nanoantenna rectifier system given by expression 5, with $Z_{rec}$ being the impedance of the rectifier and $Z_{ant}$ the input impedance of the nanoantenna. Moreover, $R_{rec}$ is the real part of the impedance of the rectifier and $R_{ant}$ the real part of the nanoantenna input impedance.

(4) $$\eta_{total}^{mat}=\frac{\int_{\lambda_{start}}^{\lambda_{stop}}P_{inc}\left(\lambda \right)\eta^{rad}\left(\lambda \right)\eta^{mat}\left(\lambda \right)d\lambda }{\int_{\lambda_{start}}^{\lambda_{stop}}P_{inc}\left(\lambda \right)\eta^{rad}\left(\lambda \right)d\lambda }$$

(5) $$\eta^{rad}=\frac{4R_{rec}R_{ant}}{|Z_{rec}+Z_{ant}{|}^{2}}$$

**Figure 6:**
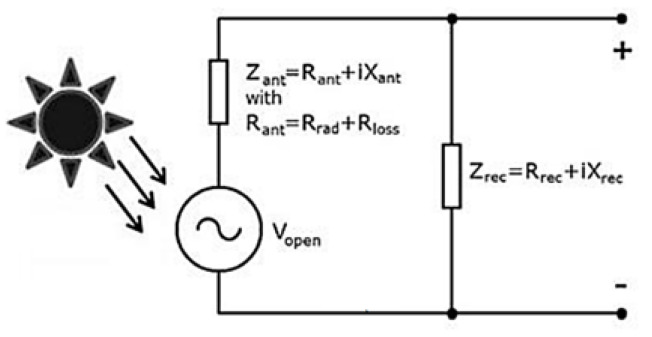
Equivalent circuit for the rectenna system (adapted from the work in [5]).

All these quantities are marked in Figure 6, an equivalent circuit of the total rectenna system, where both the transmitting and receiving processes can be easily described.

$V_{open}$ is the voltage generated by the receiving antenna at its open terminals, while $V_{rec}$ is the voltage seen at the terminals when a current is flowing to the rectifier. The useful power is the power going to the impedance of the rectifier $Z_{rec}$ and it is given by expression 6.

(6) $$P_{rec}=\frac{R_{rec}}{2}\frac{V_{open}^{2}}{|Z_{rec}+Z_{ant}{|}^{2}}$$

This power is maximal under optical matching conditions, i.e., $Z_{rec}=Z_{ant}*$, leading to expression 7.

(7) $$P_{rec}=\frac{V_{open}^{2}}{8R_{ant}}$$

Finally, to define the total rectenna efficiency, $\eta_{total}^{rec}$, presented on expression 8, is just needed to sum expressions 1 and 4.

(8) $$\eta_{total}^{rec}=\eta_{total}^{rad}\eta_{total}^{mat}$$

## 6. Model: Solar Cell

**Figure 7:**
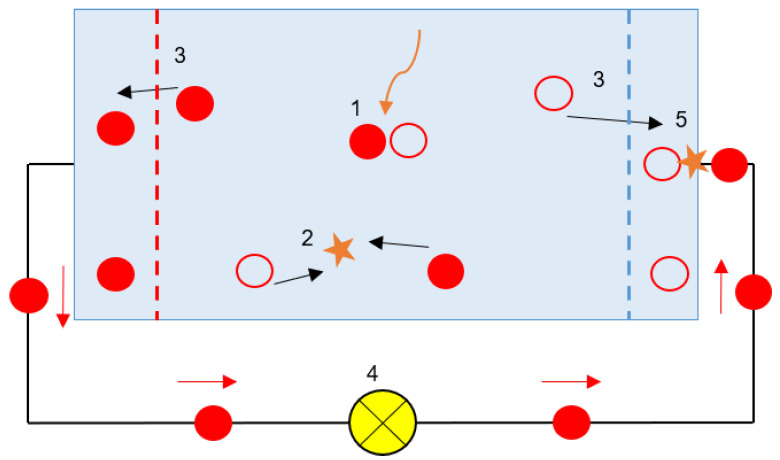
Simple model a solar cell connected to a load (sourced from the work in [43]).

A solar cell, shown in Figure 7, is a PIN structure device with no voltage directly applied across the junction. The solar cell converts light into electrical power and delivers this power to a load. This process requires a material that can absorb the light photons. The interaction of an electron with a photon leads to the promotion of an electron from the valence band into the conduction band leaving behind a hole, i.e., the absorption of a photon by a semiconductor material results in the generation of an electron–hole pair. After an electron–hole pair is created, the electron and the hole move from the solar cell into an external circuit, producing a photocurrent I. The electron then dissipates its energy in the external circuit and returns to the solar cell [26,27,28,29,30,31,32,33,34,35,36,37,38,39,40,41,42].

Some processes illustrated in Figure 7 are (1) absorption of a photon leads to the generation of an electron–hole pair; (2) recombination of electrons and holes; (3) electrons and holes can be separated with semipermeable membranes; (4) the separated electrons can be used to drive an electric circuit; and (5) after all electrons passed through the circuit, they will recombine with holes.

**Figure 8:**
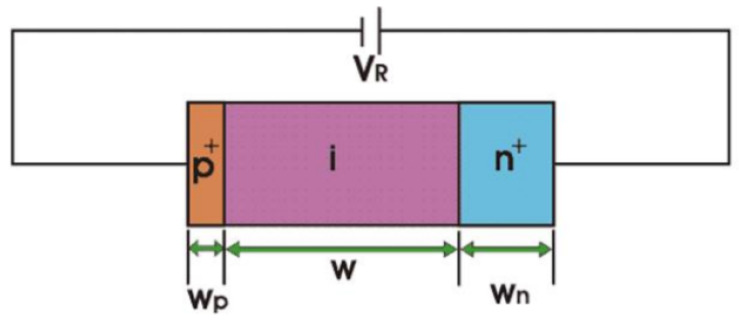
Circuit of a PIN junction, where W is the intrinsic layer width.

That solar cell is a PIN junction, also illustrated in Figure 8. The PIN structure consists of a p region and a n region separated by an intrinsic layer. The p region and n region have different electrons concentration: the n-type has an excess of electrons while the p-type has an excess of holes, i.e., positive charges. The intrinsic layer width W is much larger than the space charge width of a normal PN junction [28,29,30,31,32,33,34,35,36,37,38,39,40,41,42].

Absorption of light occurs in the intrinsic zone. A voltage $V_{R}$ is applied so that there is an electric field in the intrinsic zone large enough so when the photons are absorbed, an electron–hole pair is created, i.e., a negative charge, electron, goes to the conduction band of the semiconductor and in the valence band a positive charge is going to move on the action of the electric field [28,29,30,31,32,33,34,35,36,37,38,39,40,41,42]. Therefore, there is an electric field that immediately separates the positive from the negative charge (the negative goes to one of the terminals and the positive one goes to the other).

**Figure 9:**
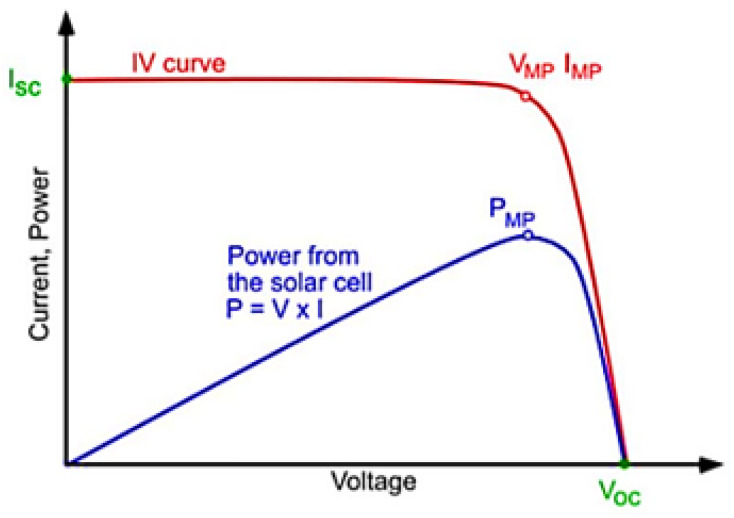
I–V curve (red) and power curve (blue) of a solar cell (sourced from the work in [45]).

The output of the PV cell is often represented with the relation between the current and voltage. This is known as the current–voltage curve (I–V curve). The I–V curve, represented in Figure 9, is a snapshot of all the potential combinations of current and voltage possible from a cell under standard test conditions (STC) [28,29,30,31,32,33,34,35,36,37,38,39,40,41,42,43,44]: (i) cell temperature: 25 °C (298.16 K); (ii) incident irradiance on the cell: G=1000$W/m^{2}$; and (iii) spectral distribution of solar radiation: AM 1.5 spectrum.

The point in the I–V curve at which the maximum power is attainable is called Maximum Power Point (MPP), being that power calculated by expression 9 [28,29,30,31,32,33,34,35,36,37,38,39,40,41,42].

(9) $$P_{MP}=V_{MP}\times I_{MP}$$

**Figure 10:**
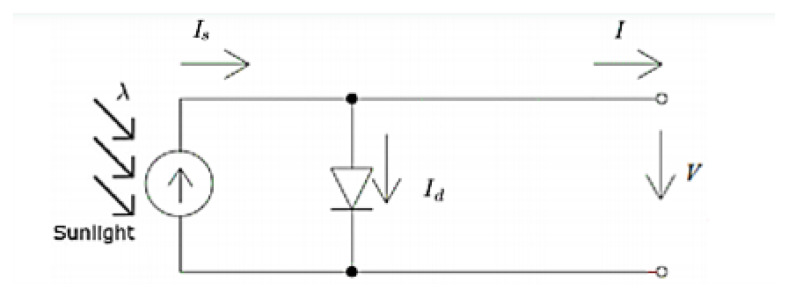
Equivalent circuit of a PV cell (1 diode and 3 parameters model-1M3P).

The representation of equipment through equivalent electrical circuits is a technique used in the field of electrical engineering. In order to study the PV equipment, a simplified electrical model is presented in Figure 10 [28,29,30,31,32,33,34,35,36,37,38,39,40,41,42].

This model has three parameters: $I_{s}$, $I_{0}$, and *n*.

$I_{s}$, also known as $I_{pv}$, represents the electric current generated by the beam of light radiation, consisting of photons, upon reaching the active surface of the cell. The level of this current depends on the irradiance [28,29,30,31,32,33,34,35,36,37,38,39,40,41,42].

The PIN junction functions as a diode that is traversed by an internal unidirectional current $I_{d}$ which depends on the voltage *V* at the terminals of the cell and on the parameters $I_{0}$ and *n*, as it is possible to verify from expression 10 [28,29,30,31,32,33,34,35,36,37,38,39,40,41,42].

(10) $$I_{d}=I_{0}\left(e^{\frac{V}{n\;v_{T}}}-1\right)$$

Then, $I_{0}$ is the he reverse saturation current of the diode, n is the diode ideality factor and $v_{T}$ is the thermal voltage for a given temperature, determined using expression 11, from the Boltzmann’s constant, *k*, and electron charge value, *q* [28,29,30,31,32,33,34,35,36,37,38,39,40,41,42].

(11) $$v_{T}=\frac{kT}{q}$$

Using the Kirchhoff’s Current Law (KCL) on that internal node, expression 12 is revealed [28,29,30,31,32,33,34,35,36,37,38,39,40,41,42].

(12) $$I=I_{s}-I_{d}=I_{s}-I_{0}\left(e^{\frac{V}{n\;v_{T}}}-1\right)$$

**Figure 11:**
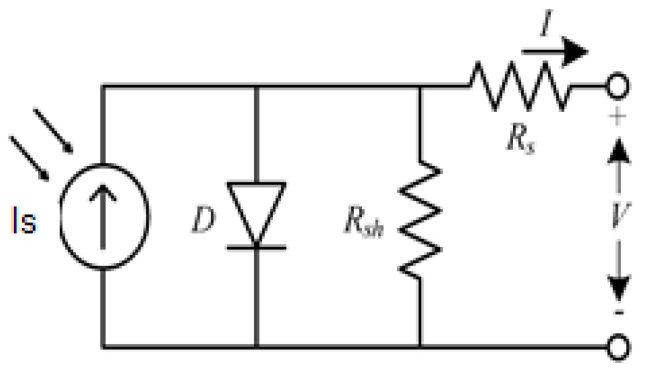
Equivalent circuit of a PV cell (1 diode and 5 parameters model-1M5P).

However, the simplified model of 1 diode and 3 parameters is not a strict representation of the PV cell. It is necessary to take into account the voltage drop in the circuit up to the external contacts, which can be represented by a series resistance $R_{s}$ and also the leakage currents, which can be represented by a parallel resistance, $R_{p}$. The influence of these parameters on the I–V characteristic of the solar cell can be studied using the equivalent circuit presented on Figure 11 [28,29,30,31,32,33,34,35,36,37,38,39,40,41,42].

The model parameters are $I_{s}$, $I_{0}$, *n*, $R_{s}$, and $R_{p}$, and thus the output current can be related to the output voltage based on expression 13 [28,29,30,31,32,33,34,35,36,37,38,39,40,41,42].

(13) $$I=I_{s}-I_{d}-I_{R_{p}}=I_{s}-I_{d}=I_{s}-I_{0}\left(e^{\frac{V}{n\;v_{T}}}-1\right)-\frac{V+R_{s}I}{R_{p}}$$

## 7. Simulation Results

In this section, a set of simulations are going to be presented. The main software used for this study was COMSOL Multiphysics®. It is generally used for modeling and simulation of real-world multiphysics systems.

First, we begin to module a PIN junction (solar cell). The purpose of the simulation is to study the propagation of light inside the semiconductor device. The incident light, an EM wave with a wavelength of 530 nm in the visible band, hits a silicon PIN junction with dimensions 150 nm, length of the p-junction; 2 um, length of the intrinsic layer; and 80 nm, length of the n-junction. The width is 0.5 um, while the PIN junction depth is 640 nm. These values are representative for a 0.35 um CMOS process.

The geometry consists of two parts: the first part is air (in gray), whose edge on top is used as the source for the EM wave that arrives to the solar cell, and the second part, in blue, is the PIN junction (from top to bottom, n-junction, intrinsic zone, and the p-junction).

The results are obtained through the simulations performed on COMSOL Multiphysics®, which uses the finite element method (FEM). This is a numerical method for solving problems of engineering and mathematical physics. To solve a problem, it subdivides a large system into smaller, simpler parts called finite elements.

In this case, FEM is used to calculate the electric field, so that the program needs to define a mesh to solve the system of equations.

**Figure 12:**
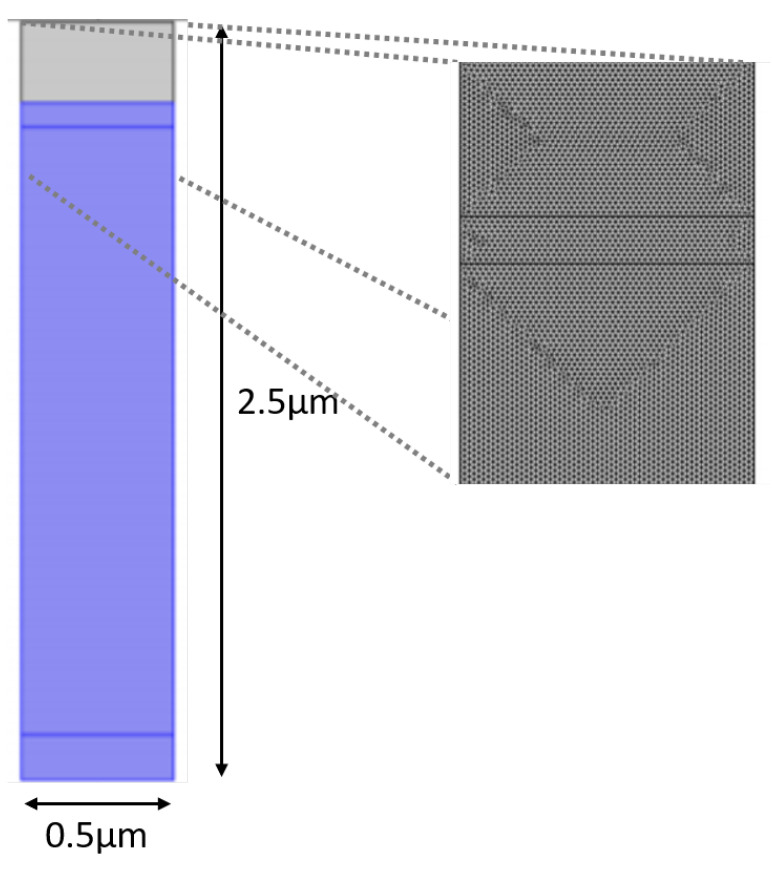
Schematic representation of a PIN junction on COMSOL Multiphysics®, as well as its mesh.

A customized mesh with triangular elements and a maximum element size of 10 nm was defined, as presented on Figure 12. The basic condition is that the mesh size should be lower than wavelength, in order not to have numerical errors in the calculation of the solution.

The parameters used for the mesh on the different simulations are represented on Table 1.

The mesh settings determine the resolution of the finite element mesh used to discretize the model. A higher value results in a finer mesh in narrow regions. In this example, because the geometry contains small edges and faces, an extremely fine mesh was designed. This will better resolve the variations of the stress field and give a more accurate result. Refining the mesh size to improve computational accuracy always involves some sacrifice in speed and typically requires increased memory usage [46].

This study is focus on the Transverse Electric (TE) polarization. TE polarized light is characterized by its electric field being perpendicular to the plane of incidence. For TE light, the magnetic field lies in the plane of incidence, thus its always perpendicular to the electric field in isotropic materials. On the other hand, Transverse Magnetic (TM) polarized light is characterized by its magnetic field being perpendicular to the plane of incidence [47].

In this case, the electric field has only one component along the z-direction (horizontal axis).

**Figure 13:**
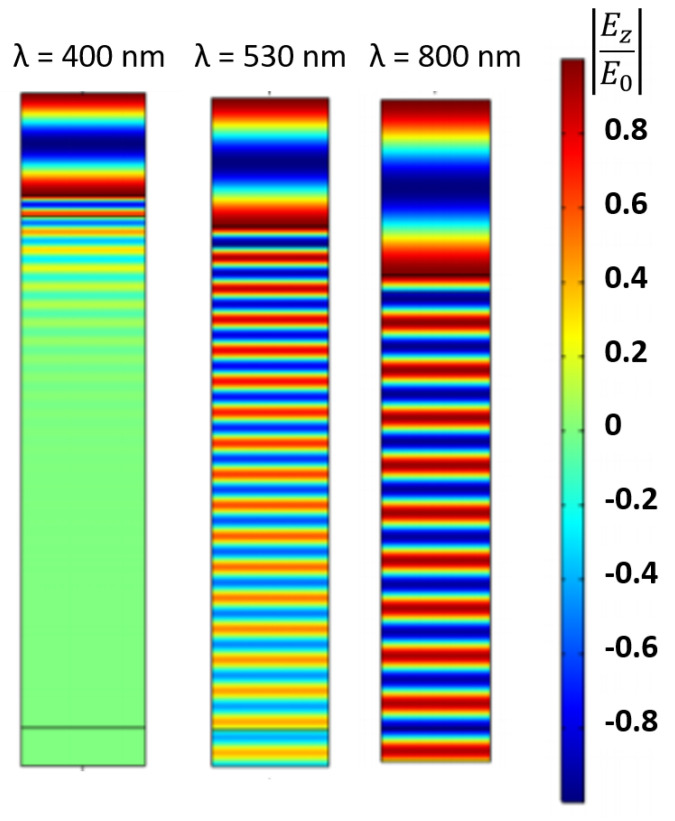
Normalized Electric field, z-component of the cross section of a PIN junction.

The PIN junction was tested for different values of λ (light wavelength): 400 nm (blue), 530 nm (green), and 800 nm (IR), as observed on Figure 13.

For a light wavelength of 400 nm, in the blue region, the photons are absorbed mainly in the top of the intrinsic region. The electric field is zero in the bottom part of the intrinsic region.

For a light wavelength of 530 nm, the electric field is stronger in the n-junction and decreases along the intrinsic zone, due to the fact that the photons are absorbed mainly in this area.

For a light wavelength of 800 nm, it is observed that the electric field practically does not decrease along the intrinsic zone. Thus, it is concluded that there is almost no absorption of photons for this wavelength.

When a nanoantenna with an array of air slits or apertures is introduced on top of the silicon PIN junction, the behavior of the electric field changes.

The main purpose of the simulations with a nanoantenna is to observe the difference between a PIN junction without nanoantenna and with a nanoantenna. Furthermore, it is our interest to analyze the evolution of the diffraction pattern as the number of air slits increases, namely, a three-slit, a seven-slit, and a fifteen-slit array, and to compare the simulation results with the results expected by the classical theory [48].

The simulation environment used is similar to that of Figure 12, where an incident light wave hits the PIN junction by propagating through the air slit arrays and absorbed along the intrinsic region.

Various experiments were performed, where the electric field was normalized to E(0), that is, the incident electric field. The incident light wave has an electric field whose amplitude is registered. This amplitude is constant for all the simulated cases and thus it will serve for normalization. It is necessary to have a normalization constant in order to better compare the electric field values for the cases when there is a nanoantenna on top of the PIN junction and when there is no nanoantenna (the structure will be different).

When light hits the surface, the electric field is no longer the incident field. It is the incident field plus the reflected field, and the reflected field varies whether or not there is a nanoantenna.

In these experiments, it was considered that the dimensions of the air slits and their spacing had subwavelength dimensions as well as the metal thickness. Furthermore, for four different values of the light wavelength, four particular cases were considered: (i) nanoantenna metal thickness, λ/10 and air slit width, λ/10; (ii) nanoantenna metal thickness, λ/100 and air slit width, λ/2; (iii) nanoantenna metal thickness, λ/100 and air slit width, λ/5; and (iv) nanoantenna metal thickness, λ/100 and air slit width, λ/10.

For each case, on top of the PIN junction a three-slit, a seven-slit, and a fifteen-slit array were tested. The procedures required to study and simulate a fifteen-slit array are identical to those used to simulate a three-slit or a seven-slit array. The parameters are the same, differing only in the number of slits. The maximum absolute values of the normalized electric field along the intrinsic region for an aluminum nanoantenna were registered on Table 2.

When the total electric field is normalized by the incident field, it is possible to immediately check whether the radiation through the intrinsic region is higher or lower than the incident radiation. In other words, if any numerical value obtained by the different simulations is greater than 1, it means that the structure itself has the capacity to transmit more light than its incidence, which indicates the occurrence of the Extraordinary Optical Transmission phenomenon. The results highlighted in green indicate the occurrence of the EOT phenomenon.

The metal thickness λ/100 proved to be more efficient and thus more simulations were performed with this size. This metal thickness was the most efficient as it can be in part attributed to the fact that aluminum, for very small film thicknesses, has a very large transmission coefficient and a low reflection coefficient. Meanwhile, for a metal thickness of λ/10 there was no occurrence of the EOT phenomenon. Contrary to what happens in the previous case, in this case practically everything is reflected and little transmitted.

The results obtained from the simulations indicate that (i) if the nanoantenna metal thickness is much smaller in relation to the wavelength, the stronger will be the electric field intensity in the intrinsic region, and (ii) the smaller the air slit width in relation to the wavelength, the smaller the intensity of the electric field in the intrinsic region, as expected given the classical theories of diffraction.

These results are confirmed by the classical theory as EOT is observed mainly due to the constructive interference of SPPs propagating between the slits of the nanoantenna, where they can be coupled from/into radiation.

The shape, dimensions, and the spacing between apertures are fundamental parameters that must be carefully sized to allow the propagation of SPPs and the occurrence of the EOT phenomenon. With the aid of MATLAB software, a 1D plot was made to compare the values of the normalized electric field along the intrinsic zone for the light wavelength of 400 nm with an aluminum nanoantenna and without nanoantennas.

It is observed in all cases that the electric field is stronger in the n-junction and then rapidly reaches the zero value in the middle of the intrinsic zone.

**Figure 14:**
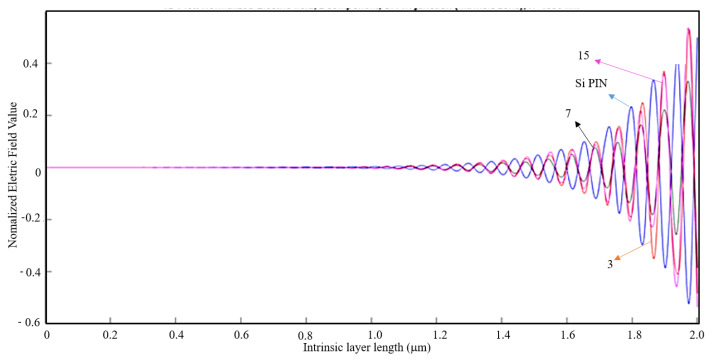
1D Plot of the normalized electric field, z-component, along the intrinsic zone for a light wavelength of 400 nm: Si PIN junction (blue); 3-slit array Al nanoantenna (red); 7-slit array Al nanoantenna (black); and 15-slit array Al nanoantenna (magenta).

By analyzing Figure 14, it is observable that the normalized electric field is stronger without nanoantennas. For this light wavelength, the results for other parameters of metal thickness and air slit width in Table 2 are quite identical, and thus for a light wavelength of 400 nm, the introduction of nanoantennas for solar harvesting does not contribute for a bigger efficiency of the solar cell.

**Figure 15:**
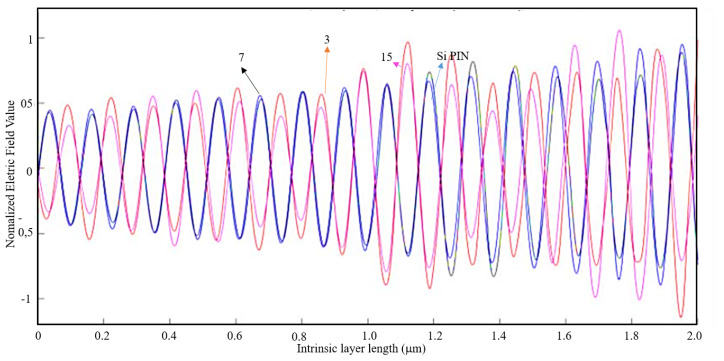
1D Plot of the normalized electric field, z-component, along the intrinsic zone for a light wavelength of 530 nm: Si PIN junction (blue); 3-slit array Al nanoantenna (red); 7-slit array Al nanoantenna (black); and 15-slit array Al nanoantenna (magenta).

Given a light wavelength of 530 nm, according to Table 2 for a metal thickness of λ/100 and an air slit width of λ/5 the EOT phenomenon barely occurs. Like in the previous case, a 1D plot was made on MATLAB and it is presented on Figure 15.

It is observable on Figure 15 that the results obtained for the normalized electric field with and without an aluminum nanoantenna are very similar. Therefore, one can conclude that for 530 nm of light wavelength the introduction of nanoantennas for solar harvesting barely contributes for a bigger efficiency of the solar cell.

For a light wavelength of 800 nm, the EOT phenomenon does not occur if the metal thickness is λ/10. For a metal thickness of λ/100, the EOT phenomenon occurs for every case and thus it is concluded that the nanoantennas are indeed efficient for this wavelength where λ/100 is the optimum thickness (see Table 2).

**Figure 16:**
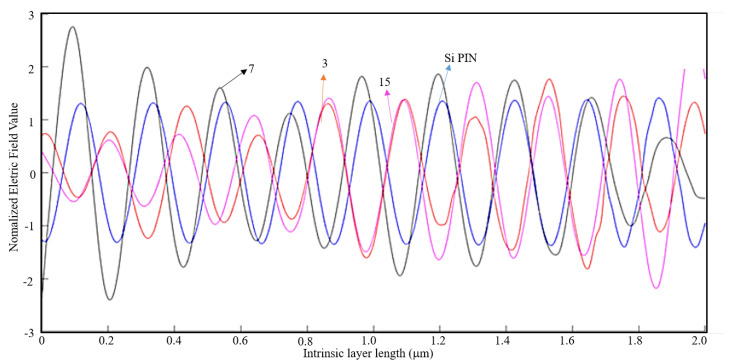
1D Plot of the normalized electric field, z-component, along the intrinsic zone for a light wavelength of 800 nm (metal thickness: λ/100; air slit width: λ/2).

**Figure 17:**
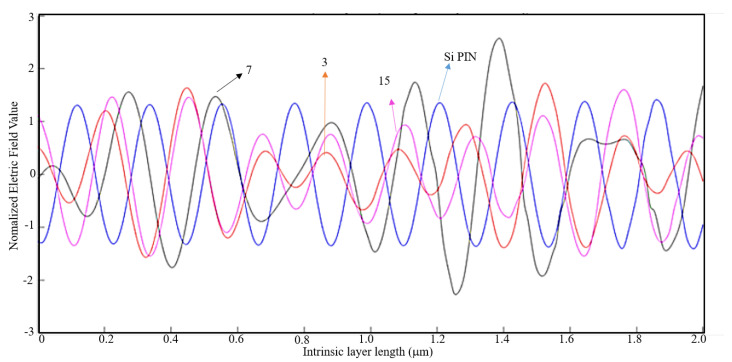
1D Plot of the normalized electric field, z-component, along the intrinsic zone for a light wavelength of 800 nm (metal thickness: λ/100; air slit width: λ/5).

**Figure 18:**
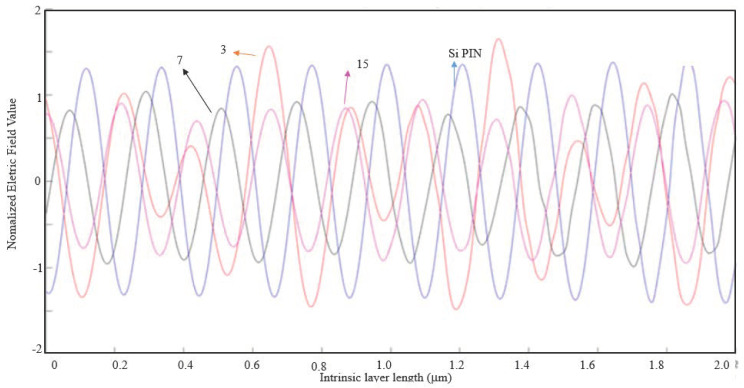
1D Plot of the normalized electric field, z-component, along the intrinsic zone for a light wavelength of 800 nm (metal thickness: λ/100; air slit width: λ/10).

Below in Figure 16, Figure 17 and Figure 18, the cases where the EOT phenomenon occurs are represented.

By analyzing Figure 16, although the 15-slit array nanoantenna has recorded the maximum absolute value of the normalized electric field, the seven-slit array is the most efficient nanoantenna type, as the normalized electric field is higher along the entire intrinsic zone.

By analyzing Figure 17, one can conclude that a seven-slit array is the most efficient along the intrinsic zone, and from Figure 18, it is concluded that it is the three-slit array.

Even though there is the occurrence of EOT, the nanoantennas are less efficient for this air slit width and thus there is no visible advantage on their implementation.

The procedures that are necessary to carry out the study and simulation of an array of slits with different material types are identical to those used previously. The metals that will be considered in the following simulations using the COMSOL Multiphysics® software are Gold (Au) and Platinum (Pt).

In Table 3 and Table 4 are registered the maximum absolute values of the normalized electric field along the intrinsic region for a nanoantenna of gold and for another of platinum, respectively, on top of a silicon PIN junction.

From the observation of both tables above, and comparing the results with an aluminum nanoantenna in Table 2, one can verify that the EOT phenomenon is present in all material types. In addition, it is possible to observe that the EOT phenomenon is stronger with an aluminum nanoantenna, as maximum absolute values of the normalized electric field along the intrinsic region of 10 × the incident field were registered for a three-slit array.

For a gold or a platinum nanoantenna, the results obtained show that the EOT phenomenon is mostly present for the light wavelengths of 800 nm and 1550 nm. These results show clear evidence of the EOT phenomenon and constitute an interesting result for the implementation of an aperture nanoantenna, as the electric field in the near-field region is strongly enhanced.

**Figure 19:**
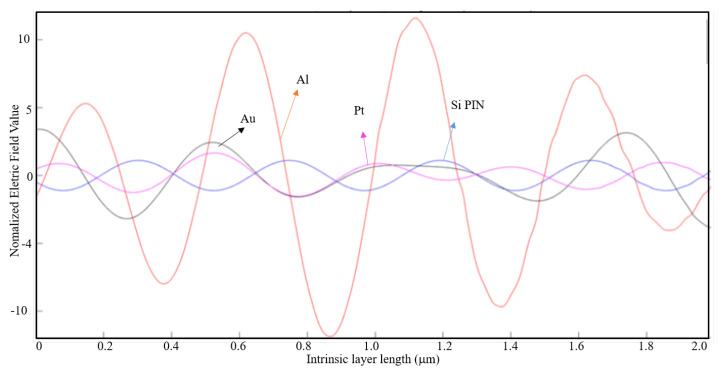
1D Plot of the normalized electric field, z-component, along the intrinsic zone for different nanoantenna material types: Si PIN junction (blue), Al nanoantenna (red), Au nanoantenna (black), and Pt nanoantenna (magenta) (3-slit array; light wavelength: 1550 nm; metal thickness: λ/100; and air slit width: λ/5).

In Figure 19, the case where the maximum absolute value of the normalized electric field along the intrinsic region for an aluminum nanoantenna on top of a Si PIN junction had the highest value, as compared with the other nanoantenna material types. From the observation of Figure 19, it is clearly visible the difference of the normalized electric field along the intrinsic zone for the aluminum nanoantenna and the other material types.

**Figure 20:**
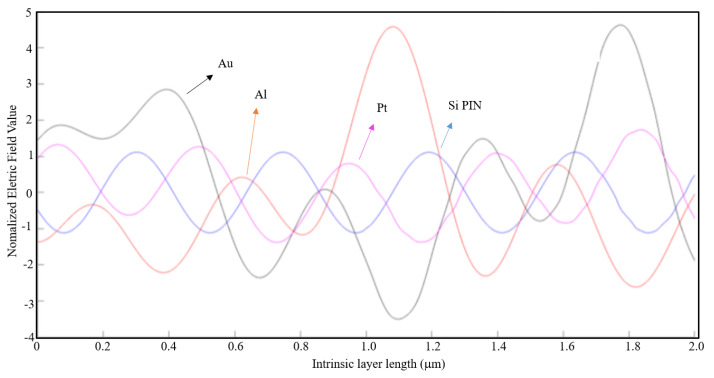
1D Plot of the normalized electric field, z-component, along the intrinsic zone for different nanoantenna material types: Si PIN junction (blue), Al nanoantenna (red), Au nanoantenna (black), and Pt nanoantenna (magenta) (7-slit array; light wavelength: 1550 nm; metal thickness: λ/100; air slit width: λ/5).

**Figure 21:**
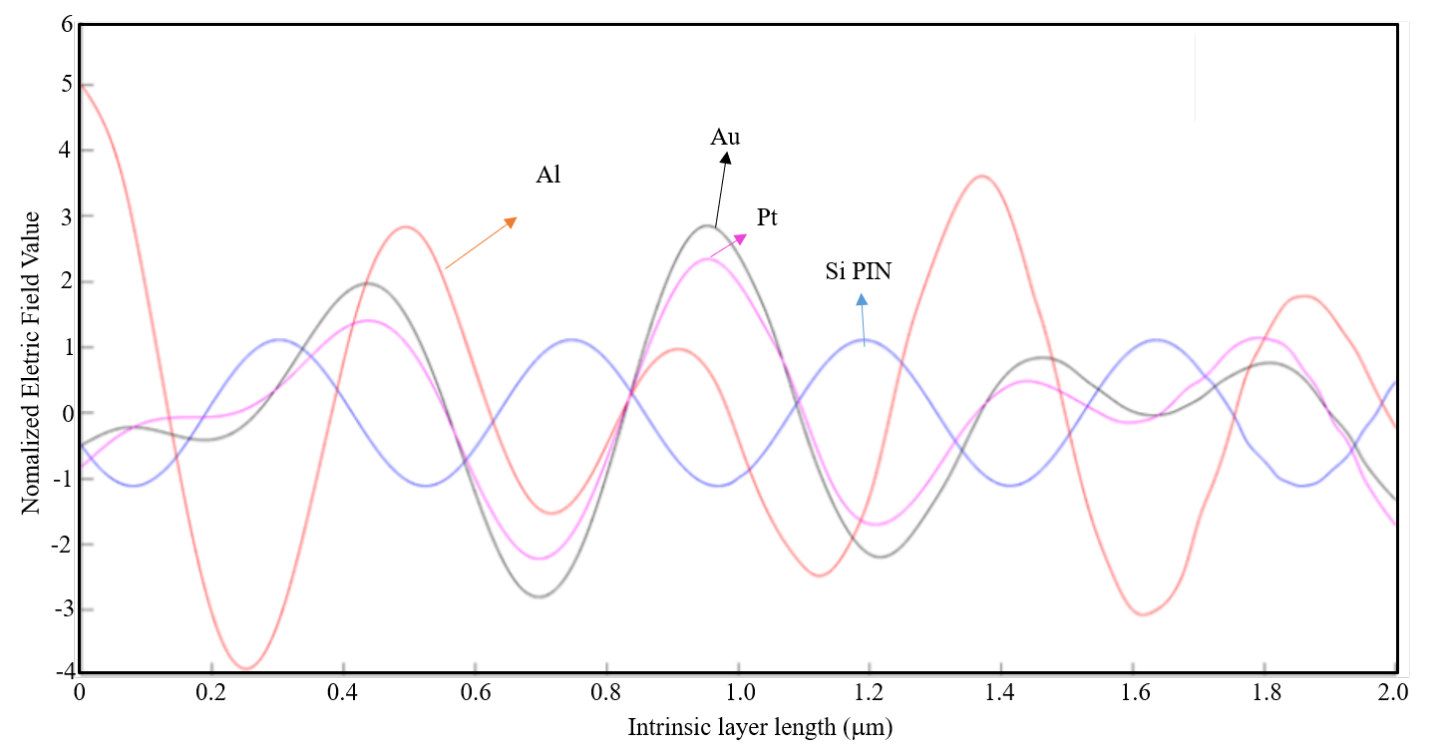
1D Plot of the normalized electric field, z-component, along the intrinsic zone for different nanoantenna material types: Si PIN junction (blue), Al nanoantenna (red), Au nanoantenna (black), and Pt nanoantenna (magenta) (15-slit array; light wavelength: 1550 nm; metal thickness: λ/100; air slit width: λ/5).

For the same parameters, in Figure 20 and Figure 21 are represented the simulation results for a seven-slit array and a fifteen-slit array nanoantenna, respectively, for the three materials types and the Si PIN junction.

By analyzing these figures, it is verified that the aluminum and the gold nanoantenna have by far a stronger normalized electric field along the entire intrinsic region compared to the platinum nanoantenna and the case without any nanoantennas. Comparing the three cases above, one can conclude that aluminum is the most appropriate material for the application of an optical antenna.

## 8. Study of the Short-Circuit Current and the Open-Circuit Voltage on the Solar Cell

As previously referred, a solar cell can be modeled using the single diode and 3 parameters model, that includes the I–V and the P–V characteristics of a typical module.

The problem of modeling a PV system is further compounded by the fact that the I–V curve of a PV module is dependent on the irradiance and temperature, which are continuously changing. Consequently, the parameters required to model a PV module must be adjusted according to the ambient temperature and irradiance [43]. Two main parameters that are used to characterize the performance of a solar cell are the short-circuit current, $I_{sc}$, and the open-circuit voltage, $V_{oc}$.

**Figure 22:**
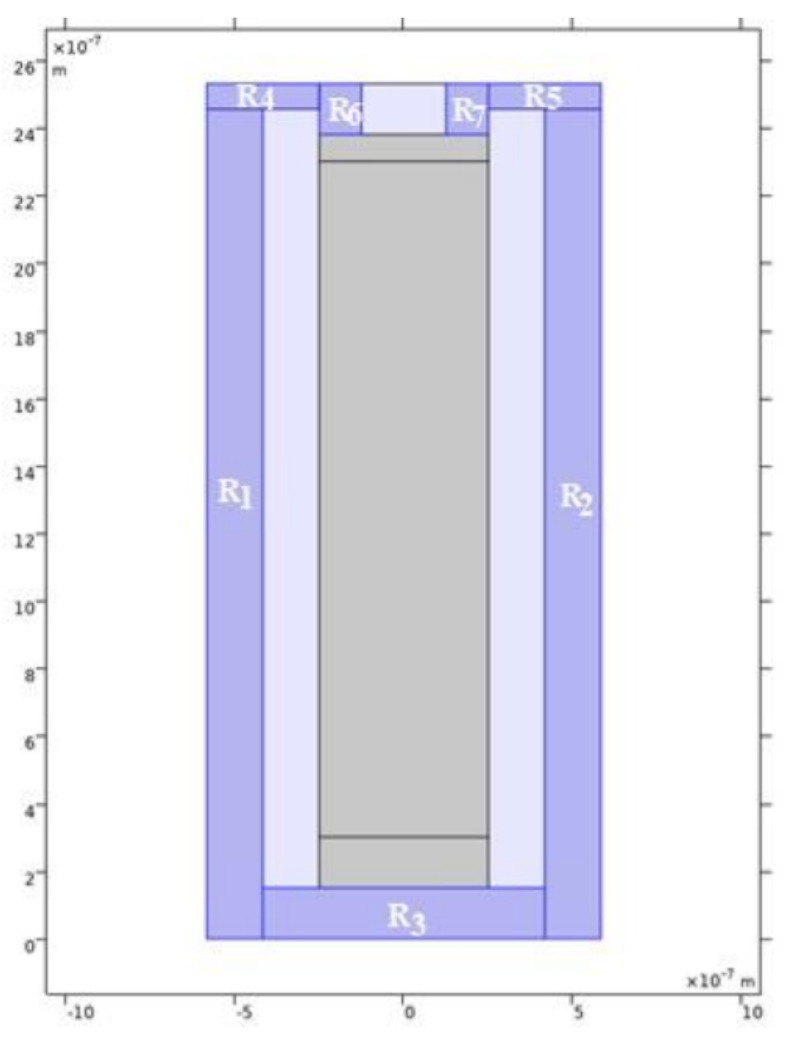
Short-circuited solar cell model (gray: Si PIN junction; dark blue: aluminum; light blue: Air).

In order to prove that the model used during the simulations on COMSOL Multiphysics® is indeed a solar cell, the short-circuit current and the open-circuit voltage were measured upon variation of the irradiance and temperature. As the software does not simulate directly the short-circuit current in the cell, a simulation of the current density norm, $J_{sc}$, was made. In this simulation, the solar cell was short-circuited as depicted in Figure 22.

According to Ibrahim, the complete equation for the short-circuit current, taking into account that it varies with the irradiance and the temperature on the solar cell, is described by expression 14, where $\alpha_{STC}$ is the thermal coefficient of the short-circuit current, measuring the variation of $I_{sc}$ with an increase of 1 °C of temperature T [49].

(14) $$I_{sc}\left(G,T\right)=\frac{G}{G_{STC}}\left[I_{sc_{STC}}+\alpha_{sc}\left(T-T_{STC}\right)\right]$$

Although COMSOL can simulate the variation in the temperature, during the simulations the temperature T on the PV cell is considered to be constant and equal to STC. Considering that the software does not simulate directly the short-circuit current in the cell, but the current density norm, given by expression 15.

(15) $$J_{sc}=\frac{G}{G_{STC}}J_{sc_{STC}}$$

Table 5 contains the average values of the current density norm with the input irradiance.

**Figure 23:**
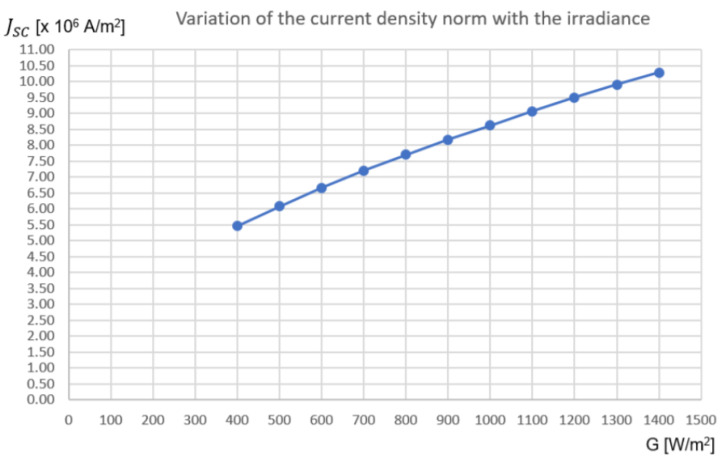
1D Plot of the variation of $J_{sc}$ with the irradiance (*x*-axis: Irradiance [W/m2]; *y*-axis: Average value of the current density norm, $J_{sc_{avg}}$ [$\times 10^{6}\;A\;m^{-1}$].

From Figure 23, the current density norm varies almost linearly with the input irradiance for this range of values on the PV cell. The slight nonlinearity can be attributed to the resistance of the material (in this case, aluminum). The resistance of any material is a function of the material’s resistivity, ρ, and the material’s dimensions, and it is given by expression 16, where L, t, and W are the length, the thickness, and the width of the material, respectively [43].

(16) $$R=\frac{\rho L}{t\;W}$$

As presented on Figure 22, there are 7 blocks or sections of aluminum surrounding the solar cell (in order to perform a short-circuit of the PV cell). Based on expression 16, it is possible to determine the value of that blocks resistance, which is presented on Table 6, for a ρ(T=25${}^{\circ }$C) = 2.70 × $10^{-8}\Omega m$ and t=640 nm.

By analyzing the values of the resistance for each block of aluminum, one can conclude that the resistance is an important factor to consider. All the values obtained for the resistance of each block are in agreement with the 0.35 um CMOS process. Therefore, the nonlinearity of the current density norm with the input irradiance is explained.

Similar to the procedure to the $I_{sc}$, it is possible to verify how $V_{oc}$ varies with the irradiance, as verified on Table 7.

The open-circuit voltage has a steady value equal to 0.8121 V, for different values of the irradiance, G, leading to the conclusion that it is not dependent on the irradiance. This value is the maximum voltage the solar cell on this model can deliver.

The open-circuit voltage varies with the irradiance by expression 17, leading to the conclusion that this variations is not very significant, because it follows a logarithmic function, where $N_{s}$ is the number of series-connected cells in a PV module (if it is a single PV cell, this value is equal to 1) and $\alpha_{oc}$ is the thermal coefficient of the open-circuit voltage.

(17) $$V_{oc}\left(G,T\right)=V_{oc_{STC}}+\frac{N_{s}k\;T}{q}ln\left(G\right)+\alpha_{oc}\left(T-T_{STC}\right)$$

To conclude, the short-circuit current, $I_{sc}$, varies nonlinearly with irradiance and its variation with temperature is fairly small depending on its temperature coefficient. When determining the dependence of the open-circuit voltage, $V_{oc}$, on temperature and irradiance, it is found that it is strongly dependent only on the temperature. It has been observed that the results obtained in this study are is accordance with what is expected by the classical theory of a photovoltaic cell and so the model that was tested on COMSOL software is valid.

## 9. Conclusions

The main objective of this article is the study and simulation of the behavior of an optical antenna with subwavelength dimensions for solar harvesting on PV panels. To perform such study, the COMSOL Multiphysics^®^ software was used, to obtain the simulation numerical results of the studied structures.

It has been demonstrated with several simulations in different conditions that the EOT phenomenon was always confirmed on nanoantennas with three materials: aluminum (Al), gold (Au), and platinum (Pt). Thus, it means that these structures have the capacity to transmit more light than its incidence, in orders of magnitude greater than predicted by standard aperture theory. These experiments provide evidence that these unusual optical properties are due to the coupling of light with SPPs on the surface of the metallic nanoantennas.

Additionally, it has been verified with the simulation results that optimum results were obtained for light wavelengths of 800 nm and 1550 nm. These results constitute an interesting result for the implementation of an aperture nanoantenna, as they cover a wide range of the spectrum: the EOT phenomenon was verified on almost the entire visible region as well as the IR region. Typical silicon solar cells have proven to be inefficient at these wavelengths.

Although most of the researchers use gold or silver to fabricate the optical antennas, the results obtained in this article show that aluminum can have even better results than the other material types, mainly due to its transmission and reflection coefficients. Furthermore, among all metals analyzed, aluminum has the smallest skin depth in the visible spectrum, as well as being cheaper than gold or platinum. However, aluminum is unstable. It oxidizes quickly, and the optical properties are lost. Therefore, aluminum has to be coated with an antioxidant compound.

## Figures and Tables

**Table 1:** Mesh parameters COMSOL Multiphysics®.

Parameter	Value
Maximum element size	1 × 10${}^{-8}$ [m]
Minimum element size	5 × 10${}^{-11}$ [m]
Maximum element growth rate	1.1
Curvature factor	0.2
Resolution of narrow regions	1

**Table 2:** Maximum absolute values of the normalized electric field along the intrinsic region.

Light Wavelength [nm]	Metal Thickness	Air Slit Width	3 Slits	7 Slits	15 Slits
400	λ/10	λ/10	0.087	0.069	0.860
400	λ/100	λ/5	0.536	0.369	0.577
400	λ/100	λ/10	0.335	0.356	0.517
530	λ/10	λ/10	0.190	0.181	0.122
530	λ/100	λ/5	1.019	1.018	1.040
530	λ/100	λ/10	0.956	0.893	0.908
800	λ/10	λ/10	0.188	0.098	0.140
800	λ/10	λ/2	1.787	2.575	2.633
800	λ/100	λ/5	1.704	2.547	1.612
800	λ/100	λ/10	1.605	1.010	1.027
1550	λ/10	λ/10	0.177	0.063	0.272
1550	λ/10	λ/2	10.053	8.071	5.806
1550	λ/100	λ/5	11.467	4.508	4.724
1550	λ/100	λ/10	2.304	1.369	1.083

**Table 3:** Maximum absolute values of the normalized electric field along the intrinsic region for a gold nanoantenna.

Light Wavelength [nm]	Metal Thickness	Air Slit Width	3 Slits	7 Slits	15 Slits
400	λ/10	λ/10	0.228	0.340	0.171
400	λ/100	λ/5	0.539	0.541	0.483
400	λ/100	λ/10	0.571	0.570	0.522
530	λ/10	λ/10	0.657	0.913	0.302
530	λ/100	λ/5	1.078	0.955	0.902
530	λ/100	λ/10	0.932	0.928	0.900
800	λ/10	λ/10	0.755	0.235	0.318
800	λ/100	λ/5	1.392	1.413	1.612
800	λ/100	λ/10	1.083	1.058	1.280
1550	λ/10	λ/10	0.045	0.076	0.207
1550	λ/100	λ/5	3.441	4.489	2.810
1550	λ/100	λ/10	1.503	1.407	2.525

**Table 4:** Maximum absolute values of the normalized electric field along the intrinsic region for a platinum nanoantenna.

Light Wavelength [nm]	Metal Thickness	Air Slit Width	3 Slits	7 Slits	15 Slits
400	λ/10	λ/10	0.130	0.223	0.152
400	λ/100	λ/5	0.584	0.578	0.569
400	λ/100	λ/10	0.572	0.578	0.969
530	λ/10	λ/10	0.210	0.411	0.197
530	λ/100	λ/5	1.013	1.002	0.994
530	λ/100	λ/10	1.215	0.957	0.968
800	λ/10	λ/10	0.191	0.139	0.145
800	λ/100	λ/5	1.588	1.591	1.243
800	λ/100	λ/10	1.261	1.048	1.105
1550	λ/10	λ/10	0.053	0.011	0.031
1550	λ/100	λ/5	1.633	1.726	2.333
1550	λ/100	λ/10	1.317	1.253	1.234

**Table 5:** Values of the current density norm with the input irradiance.

Irradiance [$W\;m^{-2}$]	$J_{{\mathrm{sc}}_{\min}}$ [$\times 10^{6}\;A\;m^{-1}$]	$J_{{\mathrm{sc}}_{\max}}$ [$\times 10^{6}\;A\;m^{-1}$]	$J_{{\mathrm{sc}}_{\mathrm{avg}}}$ [$\times 10^{6}\;A\;m^{-1}$]
400	0.88	10.05	5.47
500	0.92	11.25	6.09
600	1.00	12.33	6.67
700	1.10	13.30	7.20
800	1.20	14.20	7.70
900	1.25	15.10	8.18
1000	1.29	15.97	8.63
1100	1.35	16.78	9.07
1200	1.40	17.60	9.50
1300	1.48	18.33	9.91
1400	1.60	18.96	10.28

**Table 6:** Aluminum sections dimensions and resistance value.

Section	Length, L [m]	Width, W [m]	Resistance, R [mΩ]
$R_{1}$	$2.455\times 10^{-6}$	$1.666\times 10^{-7}$	621.447
$R_{2}$	$2.455\times 10^{-6}$	$1.666\times 10^{-7}$	621.447
$R_{3}$	$150\times 10^{-9}$	$8.335\times 10^{-7}$	7.692
$R_{4}$	$0.075\times 10^{-6}$	$3.336\times 10^{-7}$	9.485
$R_{5}$	$0.075\times 10^{-6}$	$3.336\times 10^{-7}$	9.485
$R_{6}$	$150\times 10^{-9}$	$1.250\times 10^{-7}$	50.625
$R_{6}$	$150\times 10^{-9}$	$1.250\times 10^{-7}$	50.625

**Table 7:** Values of the open-circuit voltage with the input irradiance.

Irradiance [$W\;m^{-2}$]	$V_{\mathrm{bot}}$ [V]	$V_{\mathrm{bot}}$ [V]	$V_{\mathrm{oc}}$ [V]
100	−4.5189	−5.3311	0.8121
200	−4.5189	−5.3311	0.8121
400	−4.5189	−5.3311	0.8121
800	−4.5189	−5.3311	0.8121
1000	−4.5189	−5.3311	0.8121
1200	−4.5189	−5.3311	0.8121

## Data Availability

Not applicable.
